# Patterns and clinical consequences of discontinuing HIV preexposure prophylaxis during primary care

**DOI:** 10.1002/jia2.25250

**Published:** 2019-02-15

**Authors:** Douglas Krakower, Kevin M Maloney, Victoria E Powell, Ken Levine, Chris Grasso, Kathy Melbourne, Julia L Marcus, Kenneth H Mayer

**Affiliations:** ^1^ Division of Infectious Disease Beth Israel Deaconess Medical Center Boston MA USA; ^2^ The Fenway Institute Fenway Health Boston MA USA; ^3^ Department of Population Medicine Harvard Medical School and Harvard Pilgrim Health Care Institute Boston MA USA; ^4^ Department of Epidemiology Emory University Atlanta GA USA; ^5^ Gilead Sciences Foster City CA USA

**Keywords:** HIV, PrEP, primary care, discontinuations, adherence, community health center, men who have sex with men

## Abstract

**Introduction:**

Discontinuations of HIV preexposure prophylaxis (PrEP) by at‐risk individuals could decrease the effectiveness of PrEP. Our objective was to characterize patterns of, reasons for, and clinical outcomes associated with PrEP discontinuations in primary care.

**Methods:**

We conducted medical chart reviews for patients prescribed PrEP during 2011 to 2014 at a Boston community health centre specializing in healthcare for sexual and gender minorities. Patients were followed through 2015. We characterized patients’ sociodemographics, relationship status, behavioural health conditions, patterns of and reasons for PrEP discontinuations, and HIV seroconversions. Cox proportional hazards models were used to assess patient factors associated with PrEP discontinuations.

**Results:**

Of the 663 patients prescribed PrEP, the median age was 33 years, 96% were men who have sex with men (MSM) and 73% were non‐Hispanic white; 40% were in committed relationships and 15% had HIV‐infected partners. Patients either used PrEP continuously (60%), had 1 or more discontinuations (36%), or did not initiate PrEP (4%). Discontinuations were most often due to a decrease in HIV risk perception (33%), non‐adherence to care plans (16%), or insurance barriers (12%). Of the 7 (1.1%) PrEP patients diagnosed with HIV, 1 was HIV‐infected at baseline, 2 seroconverted while using PrEP, and 4 seroconverted after discontinuations. In a multivariable model adjusted for race/ethnicity, relationship status, substance use disorders, and insurance status, those who were less than 30 years old (aHR 2.0, 95% CI 1.4 to 2.9 for ages 18 to 24, aHR 2.2, 95% CI 1.6 to 3.1 for ages 25 to 29, vs. ages 30 to 39 years), who identified as transgender women (aHR 2.0, 95% CI 1.2 to 3.4, vs. cisgender men), and who had mental health disorders (aHR 1.2, 95% CI 1.1 to 1.4 for each additional disorder) were more likely to have discontinuations.

**Conclusions:**

Discontinuations of PrEP use among this American sample of predominately MSM were common, particularly among patients who were younger, identified as transgender women, or had behavioural health issues. As HIV seroconversions occurred after discontinuations of PrEP, strategies to prevent inappropriate discontinuations are needed.

## 1 Introduction

As there are nearly 40,000 new HIV infections each year in the US 1, there is a need to implement and optimize effective HIV‐prevention strategies. Randomized‐controlled studies have demonstrated that daily and event‐driven HIV preexposure prophylaxis (PrEP) using tenofovir disoproxil fumarate with emtricitabine (TDF‐FTC) are safe and can decrease rates of new HIV infections among individuals at risk for HIV infection when taken with high adherence 2, 3, 4, 5, 6. Observational studies suggest that PrEP provision in healthcare settings can reduce HIV incidence for populations with high rates of HIV infections, including men who have sex with men (MSM) 7, 8. Scale‐up of PrEP as part of combination prevention is a priority in HIV‐prevention 9.

However, discontinuations of PrEP use among individuals with ongoing risks for acquiring HIV could decrease the effectiveness of PrEP 10, 11. Observational studies of PrEP prescribing in healthcare settings have demonstrated that many patients who initiate PrEP subsequently discontinue its use 12, 13, 14, 15. Within an integrated healthcare system in Northern California, discontinuations of PrEP use have been associated with incident HIV diagnoses 11. These findings suggest that proactively identifying individuals at risk for discontinuing PrEP and providing them with support to continue PrEP might increase its effectiveness. The development of effective strategies to support PrEP continuation, although, will depend on a deeper understanding of the reasons for discontinuation. Some individuals might intentionally discontinue using PrEP during periods when they perceive themselves to be at lower risk for HIV infection (e.g. during periods of abstinence or when entering monogamous sexual relationships with HIV‐uninfected partners or HIV‐infected partners with viral suppression) 16. Others may discontinue PrEP due to insurance or financial barriers or because of psychosocial challenges to persisting with PrEP (e.g. mental health or substance use disorders). Few studies have examined in detail the patterns and context of PrEP discontinuations, including how often discontinuations occur and whether there are patient or structural factors that are associated with discontinuations.

Our study objective was to characterize patterns of and reasons for PrEP discontinuations among individuals prescribed PrEP during primary care. We therefore conducted a comprehensive review of electronic health records (EHR) data for all patients prescribed PrEP from 2011 through 2014 at Fenway Health, a community health centre in Boston that specializes in healthcare for sexual and gender minorities and is the largest PrEP provider in New England.

## 2 Methods

### 2.1 Study population

The study cohort included all patients prescribed PrEP at Fenway Health from 26 January 2011, when the first PrEP prescription was written at Fenway Health, through 31 December 2014, with follow‐up through 31 December 2015. Cohort inclusion criteria included: (1) aged 18 years or older; (2) receipt of TDF/FTC prescription for greater than 28 days and without prescriptions for additional antiretroviral medications, to exclude use of TDF/FTC for postexposure prophylaxis; and (3) HIV‐uninfected status at the time of TDF/FTC prescription, defined as no HIV diagnosis code or positive HIV test result (i.e. any HIV antibody or antibody/antigen assay, or HIV RNA).

### 2.2 Data collection

Fenway Health uses the Centricity™ Electronic Medical Record (GE Healthcare, Boston, MA) for clinical documentation. Structured EHR data for variables pertinent to PrEP care were collected using Structured Query Language searches. Non‐structured EHR data were collected through comprehensive reviews of clinicians’ visit notes, emails and telephone communication by a research assistant using a standardized case report form developed for this study.

### 2.3 Study measures

Data were collected on patients’ sociodemographics, relationship status, behavioural health comorbidities, hepatitis B status, PrEP use and discontinuations, and HIV seroconversions. Sociodemographics were extracted from registration data and included age, sex assigned at birth, gender identity, race, ethnicity, and insurance status at the time of first PrEP prescription. Relationship status was based on clinician documentation and characterized as single versus being in a committed relationship; patients without documented partnership status (1%) were categorized as single. Behavioural health conditions included mental health diagnoses, including depression, anxiety, attention deficit disorders, post‐traumatic stress disorder (PTSD), schizophrenia, bipolar disorder, and substance use disorders, including alcohol use disorders and recreational drug use. Mental health and substance use disorders were based on patients’ medical problem lists and diagnosis codes. We only assigned mental health and substance use diagnoses to patients referred for, or receiving, treatment for these conditions to increase specificity for these diagnoses. Hepatitis B status was defined by documentation of laboratory test results, patients’ self‐report of their hepatitis B status to their provider, or providers’ summaries of external health records.

For PrEP use, we recorded dates of initiation, any discontinuations, and any re‐initiations. Discontinuations were defined as interruptions in PrEP use for greater than seven days. Dates of initiation and re‐initiation were defined by prescription dates unless clinicians documented alternative dates, such as after patient‐reported delays in PrEP initiation after receiving prescriptions. Date of discontinuation was defined by documentation in chart notes by clinicians or patients (e.g. patient emails to clinicians). When discontinuation dates were not clearly documented, they were estimated using the midpoint between the start and end dates for the last PrEP prescription (e.g. 45 days after their last prescription for a 90‐day prescription). Person‐time of PrEP use was calculated, with accrual beginning at PrEP initiation and ending at PrEP discontinuation, loss to follow‐up, or end of study period. Patients were categorized as lost to follow‐up if they did not renew their PrEP prescription, had no documented reason for PrEP discontinuation, and did not receive subsequent care at Fenway Health through 2015. Patients accrued additional person‐time if PrEP was re‐initiated after discontinuation.

We assigned reasons for discontinuations based on clinician documentation. Reasons included decreased self‐perceived risk of HIV acquisition, patient preference, non‐adherence to care plans, medication intolerance, medication‐related toxicities, insurance barriers, financial barriers, loss to follow‐up, transfer of care outside of Fenway Health with intent to discontinue PrEP, and discontinuation without a documented reason. Non‐adherence to care plans was defined as clinician discontinuation of PrEP due to patients’ lack of attendance at follow‐up visits, suboptimal medication adherence (as defined by clinician assessment and documentation of non‐adherence), or use of PrEP medications other than as prescribed.

HIV seroconversions were defined as positive HIV laboratory testing after PrEP prescriptions with confirmatory documentation by clinicians. We examined timing of seroconversions relative to PrEP prescription, patient initiation and any discontinuations. HIV drug resistance data after seroconversions were extracted from clinical HIV genotype tests.

A study investigator independently reviewed EHRs for approximately 5% of study patients and discussed discrepancies in completion of case report forms with the research assistant until consensus was achieved.

### 2.4 Analyses

Cox proportional hazards models were used to assess factors associated with time to PrEP discontinuations. Patients without discontinuations were censored at HIV seroconversion, when transferring care to other clinics with intent to continue PrEP, or at the end of the study period. Each time interval of PrEP use was included in our models, unordered, with robust sandwich variance estimators used to account for within‐subject correlation of observations. The proportional hazards assumption was assessed using time‐dependent covariates in final models. Independent variables for bivariable models were selected *a priori* using clinical judgement. Age, gender, race and ethnicity, relationship status, substance use disorder, and insurance status were defined categorically. Number of mental health disorders was continuous. All variables in unadjusted models were used in final adjusted models.

We performed sensitivity analyses to assess potential misclassification bias by re‐fitting models with the following adjustments: reclassification of loss to follow‐up as censored (rather than as a discontinuation) and exclusion of patients with indeterminate relationship status (rather than defining them as single). For patients who had uncertain discontinuation dates, we performed sensitivity analyses by recalculating person‐time for PrEP using: (1) their last documented date of PrEP use (rather than using their last documented date of PrEP use plus half the number of doses remaining on their last prescription); and using (2) their last documented date of PrEP use plus the full number of doses remaining on their last prescription.

Study procedures and a waiver of informed consent were approved by Fenway Health's Institutional Review Board.

## 3 Results

### 3.1 Sociodemographics

There were 663 unique patients who were prescribed PrEP and followed through the end of 2015. These patients contributed 743 person‐years of PrEP use with a median of 1.2 years per person (interquartile range (IQR) 0.6, 1.6). The median age was 32.6 years (IQR 27.3, 43.2); 96% of the sample identified as male and 3% as transgender women, and 73% were non‐Hispanic White and 7% were Black. Forty percent of patients were in committed relationships at first PrEP prescription, and 15% of the overall cohort had HIV‐infected partners. Mental health and substance use disorders were common, with 37% of patients having been diagnosed with anxiety, 36% with depression, 16% with attention disorders, 11% with recreational drug use disorders, 11% with alcohol use disorders, 5% with PTSD, and 4% with bipolar disease or schizophrenia. Most patients (84%) had private insurance, while 13% had Medicaid, 2% had Medicare, and 1% were uninsured. A majority of patients (65%) were immune to hepatitis B; only 1 patient had chronic hepatitis B infection (Table 1).

**Table 1:** Characteristics of patients who were prescribed HIV preexposure prophylaxis

Characteristic	All Patients who were Prescribed PrEP (N = 663)
Age, at first PrEP prescription (n, %)	
18 to 24	88 (13.3)
25 to 29	168 (25.3)
30 to 39	208 (31.4)
40+	199 (30.0)
Gender (n, %)	
Male	636 (95.9)
Female	3 (0.5)
Transgender female or trans‐feminine identifying	20 (3.0)
Transgender male or trans‐masculine identifying	4 (0.6)
Race and ethnicity (n, %)	
White (non‐hispanic)	481 (72.6)
Black	43 (6.5)
Asian or Pacific Islander	24 (3.6)
Multiracial/other	61 (9.2)
Hispanic or Latinx	44 (6.6)
Missing	10 (1.5)
Relationship status, at first PrEP prescription (n, %)	
Single^a^	398 (60.0)
Committed relationship	265 (40.0)
HIV‐infected partner	100 (15.1)
HIV‐uninfected partner/Not documented	165 (24.9)
Employment status (n, %)	
Full‐time	517 (78.0)
Part‐time	8 (1.2)
Unemployed	21 (3.2)
Student	72 (10.9)
Disabled	8 (1.2)
Retired	1 (0.2)
Unknown	36 (5.4)
Mental health disorder, ever^b^ (n, %)	
Anxiety	247 (37.3)
Depression	240 (36.2)
Attention deficit disorder^c^	106 (16.0)
Post‐traumatic stress disorder	33 (5.0)
Bipolar disorder or schizophrenia	24 (3.6)
Substance use disorder, ever (n, %)	
Non‐alcohol recreational drug^b^	72 (10.9)
Crystal methamphetamine	52 (7.8)
Cocaine	23 (3.5)
Gamma‐hydroxybutyric acid	11 (1.7)
Alcohol	73 (11.0)
Marijuana	28 (4.2)
Injection drug use, ever (n, %)	28 (4.2)
Insurance status, at first PrEP prescription^b^ (n, %)	
Private	559 (84.3)
Medicaid	84 (12.7)
Medicare	13 (2.0)
No insurance	5 (0.8)
Other	27 (4.1)
Hepatitis B status^d^	
Immune	430 (64.9)
Susceptible	159 (24.0)
Isolated positive hepatitis B core antibody	7 (1.1)
Chronic hepatitis B infection^e^	1 (0.2)
Not documented	66 (10.0)

PrEP, preexposure prophylaxis; SD, standard deviation. ^a^Includes divorced, formal separation, widowed, and any patient without documentation of committed relationship. ^b^Categories not mutually exclusive. ^c^Includes attention deficit disorder and attention deficit hyperactivity disorder. ^d^Patients with positive hepatitis B surface antibody with or without positive hepatitis B core antibody were categorized as immune to hepatitis B. ^e^This patient did not experience an acute exacerbation of chronic hepatitis B during the study period.

### 3.2 PrEP use and discontinuations

**Figure 1:**
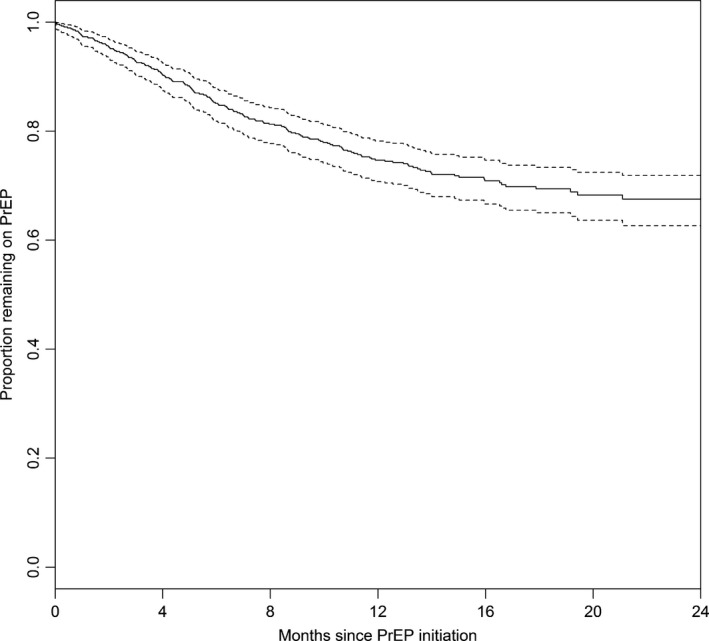
Kaplan–Meier curve of time to first discontinuation among patients who initiated HIV preexposure prophylaxis (PrEP) (n = 635). Dotted lines indicate 95% confidence intervals.

Of the 663 patients prescribed PrEP, 96% were documented to have initiated PrEP, while 4% did not initiate or were lost to follow‐up after the first prescription. A majority of patients (60%) used PrEP continuously after initiation and 36% had one or more discontinuations, with 28% having one discontinuation, 6% having two discontinuations, 1% having three discontinuations, and <1% having four discontinuations. The median time to first discontinuation was 4.1 months (IQR 2.0, 8.5) (Figure 1). Nearly one‐fifth of the cohort (18%) discontinued PrEP and did not reinitiate, 15% discontinued once and re‐initiated, 3% discontinued twice and re‐initiated each time, and 1% discontinued three times and re‐initiated each time.

In total, there were 302 PrEP discontinuation events (Table 2). The most common reasons for discontinuations were decreased HIV risk perception (33% of all discontinuations), non‐adherence to care plans (16%), and insurance barriers (12%). Of the discontinuation events attributable to non‐adherence to care plans, 71% were due to lack of attendance at follow‐up visits, 25% were due to non‐adherence to medications, and 4% were due to using PrEP medications other than as prescribed. About 14% of discontinuations did not have a documented reason. Only 3% (n = 9) of all discontinuation events were due to medication‐related toxicities: 2 of these were due to changes in kidney function (both of which normalized during the study period) and 7 were due to elevated liver enzymes (five of which normalized). No toxicities were associated with serious end‐organ disease.

**Table 2:** Reasons for and patterns of HIV preexposure prophylaxis discontinuation events

Reasons for discontinuation	First discontinuation (N = 239) n (%)	Second discontinuation (N = 51) n (%)	Third discontinuation (N = 10) n (%)	Fourth discontinuation (N = 2) n (%)	Total discontinuations (N = 302) n (%)
Decrease in HIV risk perception	84 (35.2)	13 (25.5)	1 (10.0)	1 (50.0)	99 (32.8)
Non‐adherence to care plan^a^	34 (14.2)	11 (21.6)	2 (20.0)	1 (50.0)	48 (15.9)
Not documented	27 (11.3)	12 (23.5)	3 (30.0)	—	42 (13.9)
Insurance barrier	30 (12.6)	6 (11.8)	1 (10.0)	—	37 (12.3)
Medication intolerance	14 (5.9)	3 (5.9)	—	—	17 (5.6)
Loss to follow‐up	13 (5.4)	—	1 (10.0)	—	14 (4.6)
Other	12 (5.0)	3 (5.9)	—	—	15 (5.0)
Medication related toxicities	9 (3.8)	—	—	—	9 (3.0)
Financial barrier	7 (2.9)	—	—	—	7 (2.3)
Patient preference^b^	6 (2.5)	3 (5.9)	1 (10.0)	—	10 (3.3)
HIV seroconversion^c^	2 (0.8)	—	—	—	2 (0.7)
Transfer of care with intent to discontinue^d^	1 (0.4)	—	1 (10.0)	—	2 (0.7)

^a^Includes non‐adherence to clinical visits, laboratory monitoring and/or medications, or using medications other than as prescribed. ^b^Includes expressed preference to discontinue PrEP and “experiencing stigma from romantic partners, sexual partners, and/or peers.” ^c^Of the seven seroconversions in the overall cohort, four patients discontinued PrEP prior to seroconversion and their discontinuation events were attributed to other causes, and one patient acquired HIV prior to being prescribed PrEP and was excluded from analyses. ^d^Both of these patients were moving to a country where PrEP was not available but indicated interest in continuing PrEP, if it had been available.

### 3.3 HIV seroconversions

Seven patients were diagnosed with HIV after PrEP prescriptions (Table 3). One patient had positive HIV antibody/antigen and HIV RNA tests on the day PrEP was prescribed and never initiated PrEP. Four patients seroconverted after discontinuing PrEP, for the following reasons: one patient was experiencing PrEP‐related stigma after being criticized by his partner for using PrEP (diagnosed with HIV 10.5 months after discontinuation); one had uncontrolled mental illness (diagnosed seven months after discontinuation); one experienced insurance barriers (diagnosed 18 months after discontinuation); and one discontinued after decreased HIV risk perception (diagnosed seven months after discontinuation). Two patients seroconverted while using PrEP. One of those two patients had a clinical history suggesting PrEP may have been started during acute HIV infection and had resistance mutations (K65R/M184V) associated with ineffective tenofovir and emtricitabine use on genotype testing. The second of those patients had a high‐risk exposure after baseline negative HIV antibody/antigen testing but before initiating PrEP and may also have initiated PrEP after HIV acquisition, and had an M184V mutation; suboptimal adherence to PrEP was reported, so timing of infection in relation to PrEP use was difficult to determine.

**Table 3:** HIV seroconversions among patients prescribed HIV preexposure prophylaxis

Patient	Year of initial PrEP prescription	Age at HIV diagnosis, years	Gender	Estimated timing of HIV acquisition	Time to seroconversion after discontinuation (Days)	Clinical circumstances	HIV drug resistance mutations
Patient 1	2012	44	Male	Before or within five months after initiating PrEP	N/A	Prescribed PrEP one month after negative HIV antibody/antigen assay. Clinical history suggests PrEP was probably initiated after undiagnosed HIV infection.	K65R, M184V
Patient 2	2012	31	Male	After discontinuing PrEP	544	Discontinued PrEP because of insurance barriers.	None
Patient 3	2013	25	Male	After discontinuing PrEP	224	Discontinued PrEP due to decreased HIV risk perception.	None
Patient 4	2013	28	Male	Before initiating PrEP	N/A	Prescribed PrEP one month after negative HIV antibody/antigen assay. Had positive HIV antibody/antigen assay and HIV RNA test on day that PrEP was prescribed. Did not initiate PrEP.	None
Patient 5	2014	40	Male	After discontinuing PrEP	315	Discontinued PrEP because of concerns about PrEP‐related stigma.	None
Patient 6	2014	25	Male	After discontinuing PrEP	216	Discontinued PrEP because of uncontrolled behavioural health conditions.	None
Patient 7	2014	27	Male	Before or within six months after initiating PrEP	N/A	Prescribed PrEP one week after negative HIV antibody/antigen assay. Patient reported possible HIV exposure within three weeks prior to PrEP initiation. Used PrEP with suboptimal adherence. Timing of seroconversion relative to PrEP use indeterminate.	M184V

N/A, not applicable; K65R, amino acid shift from lysine to arginine at position number 65; M184V, amino acid shift from to methionine to valine at position number 184; RNA, ribonucleic acid.

### 3.4 Factors associated with discontinuations

There were 602 patients included in the Cox proportional hazards models after excluding 61 patients for the following reasons: non‐initiation of PrEP or loss to follow‐up after their first PrEP prescription (n = 28); insurance status of “none” or “other” (n = 26); missing information on race and ethnicity (n = 10); or HIV acquisition before initiating PrEP (n = 1).

In unadjusted models, time to discontinuation was associated with younger age, identifying as transgender female, increasing number of mental health disorders, alcohol use disorder and recreational drug use disorders, and having public insurance (Table 4). Having an HIV‐uninfected partner was inversely associated with time to discontinuations. In a model adjusted for race/ethnicity, relationship status at first PrEP prescription, substance use disorders, and insurance status, younger age (adjusted hazard ratio (aHR) 2.0, 95% confidence intervals (CI) 1.4 to 2.9 for ages 18 to 24 and aHR 2.2, 95% CI 1.6 to 3.1 for ages 25 to 29 years, vs. ages 30 to 39 years), identifying as transgender female (aHR 2.0, 95% CI 1.2 to 3.4, vs. cisgender male), and increasing number of diagnosed mental health disorders (aHR 1.2, 95% CI 1.1 to 1.4 for each additional mental health disorder) were associated with time to discontinuations (Table 4). The proportional hazards assumption was met.

**Table 4:** Factors associated with time to any discontinuation of HIV preexposure prophylaxis (n = 602)

Covariate	No Disc (N = 376)	1 Disc (N = 178)	2 Disc (N = 40)	3 Disc (N = 6)	4 Disc (n = 2)	Any disc	
						HR (95% CI)	aHR (95% CI)
Age (n, %)							
18 to 24	37 (9.8)	28 (15.7)	10 (25.0)	2 (33.3)	**–**	**2.2 (1.5, 3.2)**	**2.0 (1.4, 2.9)**
25 to 29	69 (18.4)	58 (32.6)	19 (47.5)	2 (33.3)	1 (50.0)	**2.2 (1.6, 3.1)**	**2.2 (1.6, 3.1)**
30 to 39	130 (34.6)	51 (28.7)	5 (12.5)	1 (16.7)	1 (50.0)	Ref	Ref
40+	140 (37.2)	41 (23.0)	6 (15.0)	1 (16.7)	–	0.8 (0.5, 1.1)	0.8 (0.5, 1.2)
Gender (n, %)							
Cisgender male	367 (97.6)	166 (93.3)	36 (90.0)	6 (100.0)	2 (100.0)	Ref	Ref
Cisgender female	2 (0.5)	–	–	–	–	—	—
Transgender female	5 (1.3)	11 (6.2)	3 (7.5)	–	**–**	**3.0 (1.7, 5.1)**	**2.0 (1.2, 3.4)**
Transgender male	2 (0.5)	1 (0.6)	1 (2.5)	–	–	1.5 (0.5, 5.1)	0.9 (0.3, 2.5)
Race and ethnicity (n, %)							
Non‐Hispanic White	289 (76.9)	128 (71.9)	27 (67.5)	4 (66.7)	2 (100.0)	Ref	Ref
Black	20 (5.3)	13 (7.3)	4 (10.0)	1 (16.7)	–	1.5 (0.9, 2.3)	1.4 (0.9, 2.0)
Asian or Pacific Islander	10 (2.7)	7 (3.9)	2 (5.0)	–	–	1.8 (0.9, 3.4)	1.4 (0.7, 2.9)
Multiracial/other	35 (9.3)	18 (10.1)	2 (5.0)	1 (16.7)	–	1.0 (0.7, 1.6)	0.9 (0.6, 1.4)
Hispanic or Latinx	22 (5.9)	12 (6.7)	5 (12.5)	–	–	1.5 (0.9, 2.5)	1.2 (0.7, 2.2)
Relationship status (n, %)							
Single	214 (56.9)	114 (64.0)	28 (70.0)	4 (66.7)	2 (100.0)	Ref	Ref
HIV‐infected primary partner	53 (14.1)	27 (15.2)	7 (17.5)	2 (33.3)	–	0.9 (0.6, 1.3)	1.0 (0.8, 1.4)
HIV‐uninfected primary partner	109 (29.0)	37 (20.8)	5 (12.5)	–	**–**	**0.6 (0.4, 0.8)**	0.8 (0.6, 1.1)
No. of mental health disorders (mean, SD)	0.9 (1.0)	1.1 (1.1)	1.4 (1.2)	2.0 (1.1)	3.5 (0.7)	**1.3 (1.2, 1.5)**	**1.2 (1.1, 1.4)**
Substance use disorder (n, %)							
Alcohol	37 (9.8)	20 (11.2)	8 (20.0)	2 (33.3)	2 (100.0)	**1.7 (1.2, 2.4)**	1.3 (0.9, 1.9)
Non‐alcohol, recreational drug^a^	29 (7.7)	24 (13.5)	9 (22.5)	2 (33.3)	1 (50.0)	**1.9 (1.3, 2.6)**	1.2 (0.8, 1.7)
Insurance status (n, %)							
Private (any)	336 (89.4)	144 (80.9)	33 (82.5)	5 (83.3)	–	Ref	Ref
Public (Medicaid or Medicare)	40 (10.6)	34 (19.1)	7 (17.5)	1 (16.7)	2 (100.0)	**1.9 (1.4, 2.6)**	1.3 (1.0, 1.9)

Bold font indicates hazard ratios for which 95% confidence intervals do not include the value of 1. aHR, adjusted hazard ratio; CI, confidence intervals; ref, referent; Disc, discontinuation; HR, hazard ratio; SD, standard deviation. ^a^Includes drug use disorders related to cocaine, crack, crystal, GHB, heroin, poppers, ketamine, MDMA, opioids, LSD and benzodiazepine.

### 3.5 Sensitivity analyses

HRs were not affected by reclassifying loss to follow‐up as censored, by excluding patients with indeterminate relationship status, or by recalculating person‐time using the last known date of PrEP use plus all remaining prescribed doses. When reclassifying person‐time for PrEP using the last known date of PrEP use, being transgender female was no longer associated with time to PrEP discontinuation.

## 4 Discussion

In this study of the first 663 patients receiving PrEP prescriptions at a specialized community health centre, PrEP discontinuations were common, with over one‐third (36%) of PrEP patients having one or more discontinuations during the study period. Nearly one‐fifth (18%) of the cohort discontinued PrEP and did not reinitiate during this time frame. Because four of seven study patients newly diagnosed with HIV became infected after discontinuing PrEP, our study suggests a need to develop effective ways to prevent inappropriate PrEP discontinuations in care settings.

This report represents one of the first studies to use comprehensive chart reviews to characterize in detail the reasons for, and consequences of, PrEP discontinuations. In this cohort, discontinuations occurred for heterogeneous reasons. The most common reasons for discontinuing PrEP included decreased HIV risk perception, non‐adherence to care plans, insurance barriers, and medication intolerances. Other studies have also found that changes in HIV risk perception and side effects were common reasons for PrEP discontinuation 17. This heterogeneity in the reasons for discontinuing PrEP suggests that preventing inappropriate discontinuations will likely require diverse or multifaceted interventions. Interventions may include innovative forms of PrEP delivery to facilitate adherence to care plans, such as home‐based PrEP services 18, 19; pharmacist‐supported PrEP services; 20 programmes to help patients maintain insurance coverage and access financial assistance resources for PrEP care, such as through the use of health system navigators;^21^, ^22^ and strategies to increase access to mental healthcare or substance use treatment programmes. In addition, PrEP users who discontinue daily PrEP due to decreased frequency of sex might be more likely to use event‐driven PrEP without discontinuation 6, although CDC has not recommended non‐daily PrEP use 23.

In recognition of inappropriate PrEP discontinuations, Fenway Health has taken several steps to improve PrEP continuation. These include frequent trainings to ensure that providers are competent in PrEP provision, regular review of clinical protocols and reasons for HIV seroconversions among PrEP users, and implementation of health system navigators to support PrEP use. Despite trainings to standardize practice, clinicians in this study might still have differed in their criteria for non‐renewal of PrEP prescriptions (e.g. time past scheduled laboratory monitoring), which could have affected rates of discontinuations due to non‐adherence to care plans. Studies to discern optimal criteria for non‐renewal (i.e. that minimize patient burden without compromising safety) could potentially facilitate uninterrupted PrEP use.

Because many patients discontinued PrEP when perceiving decreased personal HIV risk, there is a need to develop and disseminate guidance and tools to help patients and clinicians to accurately assess risk, so they can make optimal decisions about when it might be appropriate to discontinue PrEP (i.e. during periods of lower HIV risk) consistent with prevention‐effective adherence 16. HIV risk assessment tools already exist for some populations (e.g. men who have sex with men 24, 25, 26, 27 and persons who inject drugs 28), but these tools may transfer poorly across populations 29, and further research is needed to assess the value of implementing these tools for individual assessment, especially when considering PrEP continuation or discontinuation. One patient in this study acquired HIV after discontinuing PrEP in response to PrEP‐related stigma. Thus, interventions to address stigma might also prevent deliberate but counterproductive discontinuations of PrEP. Furthermore, interventions to reengage patients who have discontinued PrEP are also needed.

We found that PrEP discontinuations were more likely to occur among persons who were younger, who identified as transgender women, and who had multiple mental health disorders. Our findings corroborates prior studies that have found an association between younger age and challenges accessing or continuing with PrEP 14, 30, 31 and highlights additional sub‐populations, including transgender women and those with mental health disorders, for whom intensive and/or tailored support for continued PrEP use may be beneficial. Because HIV incidence rates among young MSM and transgender women are among the highest in any sub‐population nationally 32, and because mental health diagnoses have been associated with HIV risk behaviours 33, 34, addressing ineffective PrEP use in these groups should be prioritized. Insurance barriers to PrEP continuation were also common, even in a location with high rates of insurance coverage (Massachusetts), suggesting that navigators, patient assistance programmes, and other strategies to overcome insurance barriers to PrEP will be critical in most jurisdictions.

The results of our study corroborate the high rates of PrEP discontinuations consistently observed in other clinical cohorts. A retrospective chart review at an academic health system in New York found that retention in PrEP care after six months was 42%. 17 In a national study of over 1000 PrEP users at the Veterans Health Administration, 44% of PrEP users discontinued PrEP within the first year 14, and 38% of PrEP users at a community‐based clinic in San Francisco discontinued PrEP by 13 months 13. Similarly, 26% of nearly 5000 patients who initiated PrEP between 2012 and 2017 at an integrated healthcare organization in Northern California discontinued PrEP 11. As all of the seroconversions among patients who were prescribed PrEP at this organization occurred after PrEP discontinuations, our study adds to the growing evidence of the negative clinical consequences of PrEP discontinuations.

In terms of other potential clinical consequences after PrEP discontinuations, we did not observe antiretroviral drug resistance mutations in clinical genotype tests among patients who seroconverted after discontinuing PrEP. Drug resistance mutations were, however, detected in patients who either initiated PrEP after undiagnosed HIV infection or who acquired HIV while using PrEP with questionable patterns of adherence. These findings suggest that efforts to prevent the emergence of drug resistance may need to focus less on PrEP discontinuations and more on rigorous approaches to ruling out undiagnosed HIV infection prior to PrEP initiation and on optimizing adherence once PrEP is initiated. We did not observe acute exacerbations of chronic hepatitis B with PrEP discontinuations, consistent with prior studies 35; however, only 1 patient in our cohort had chronic hepatitis B infection. Further studies in populations where chronic hepatitis B is more common are needed to adequately assess adverse outcomes from discontinuing PrEP among patients with hepatitis B.

Our study design has limitations. It was conducted at a community health centre specializing in healthcare for gender and sexual minorities where PrEP use was common, so our results may not be generalizable to settings where clinicians are less experienced with PrEP. These data also represent a highly insured cohort of mostly white MSM in a progressive state, so patterns and reasons for PrEP discontinuations might differ for important populations in other regions (e.g. among Black MSM in the Southern U.S.). In addition, chart reviews may result in inaccuracies when characterizing reasons for and timing of PrEP discontinuations given variability in clinicians’ assessments and documentation of this information; direct assessments with patients and clinicians to characterize discontinuations (e.g. using qualitative methods) could improve accuracy in future studies. However, comprehensive chart reviews allowed us to assess reasons for PrEP discontinuation that would not have been identifiable using only structured EHR data, and the results of our sensitivity analyses suggested that the major findings of our modelling exercises were generally robust despite potential errors in characterizing discontinuations. Finally, this study examined early adopters of PrEP, who might have differences from newer adopters, and rates of PrEP prescribing have increased at this health centre and elsewhere since 2015 36, so updated studies are needed to understand current patterns and consequences of PrEP discontinuations.

## 5 Conclusions

Over one‐third of patients prescribed PrEP at a specialized community health centre in the U.S. discontinued PrEP at least once during an average follow‐up period of approximately one year of PrEP use, and nearly one‐fifth of PrEP patients discontinued PrEP and did not reinitiate during the study period. We found that the most common reasons for discontinuations were a decrease in HIV risk perception, non‐adherence to care plans, and insurance barriers. We also found that HIV seroconversions occurred after discontinuations of PrEP. Thus, flexible and diverse interventions that can prevent inappropriate PrEP discontinuations are needed to maximize the effectiveness of PrEP.

## Competing interests

DK has received funding to complete educational content relating to HIV prevention for Medscape, MED‐IQ, DKBmed and UptoDate, Inc., and has been a consultant to Fenway Health on a research grant sponsored by Gilead. KM is an employee of and owns stock in Gilead Sciences. JM has received research grant support from Merck, and is a consultant to Kaiser Permanente Northern California on a research grant sponsored by Gilead. KHM has received unrestricted research grants from Gilead Sciences and Viiv Healthcare. KMM, VEP, KL and CG have no competing interests to declare.

## Authors’ contributions

DK, KM, KMM and KHM designed the study. KL, CG, KMM and DK completed data collection. KMM and VEP analysed the data. DK and VEP drafted the manuscript. DK, KMM, VEP, KM, JM and KHM assisted with interpretation of results. All authors reviewed and agreed to submit the manuscript.

### Funding

This work was made possible with support from an unrestricted research grant from Gilead Sciences; grants from the National Institutes of Health (NIMH MH098795, NIAID K01 AI122853); and the Harvard University Center for AIDS Research, an NIH funded programme (P30 AI060354).
